# Supplementary material for: Unveiling and understanding health inequalities: A bi-clustering study on SDG3 implementation in the Italian regions
Source: PLoS One. 2026 Mar 26;21(3):e0340438. doi: 10.1371/journal.pone.0340438 (PMC13020981; doi:10.1371/journal.pone.0340438)
Supplement: S1 Table — (DOCX) [file pone.0340438.s001.docx]

**S1 Table.** *Averaging SDG3 indicators for each Italian region*

|  | **_Abruzzo_** | **_Basilicata_** | **_Calabria_** | **_Campania_** | **_Emilia-Romagna_** | **_Friuli-Venezia Giulia_** | **_Lazio_** | **_Liguria_** | **_Lombardia_** | **_Marche_** | **_Molise_** | **_Piemonte_** | **_Provincia Autonoma di Bolzano/Bozen_** | **_Provincia Autonoma di Trento_** | **_Puglia_** | **_Sardegna_** | **_Sicilia_** | **_Toscana_** | **_Umbria_** | **_Valle d'Aosta/Vallée d'Aoste_** | **_Veneto_** |
| --- | --- | --- | --- | --- | --- | --- | --- | --- | --- | --- | --- | --- | --- | --- | --- | --- | --- | --- | --- | --- | --- |
| Alcohol (age-standardized rates) | _16.8_ | _16.4_ | _14.2_ | _12.2_ | _19.1_ | _21.5_ | _13.9_ | _17.9_ | _18.2_ | _17.2_ | _19.7_ | _18.5_ | _25.5_ | _21.4_ | _14.8_ | _19.5_ | _10.6_ | _18.7_ | _16.4_ | _24.8_ | _19.8_ |
| Influenza vaccination coverage, age 65+ | _50.5_ | _55.9_ | _57.5_ | _59.8_ | _55.4_ | _55.7_ | _53.8_ | _48.9_ | _49.1_ | _53.4_ | _55.9_ | _49.7_ | _36.7_ | _54.8_ | _56.2_ | _45.5_ | _54.1_ | _56.8_ | _65.7_ | _46.2_ | _56.9_ |
| Pediatric vaccination coverage: measles | _76.5_ | _81.1_ | _78.7_ | _68.9_ | _81.1_ | _77.6_ | _81.3_ | _77.8_ | _82.6_ | _77.5_ | _76.9_ | _82.2_ | _63.1_ | _79.8_ | _79.8_ | _81.1_ | _76.9_ | _81.9_ | _82.0_ | _77.0_ | _80.7_ |
| Pediatric vaccination coverage: polio | _85.7_ | _86.3_ | _84.6_ | _72.9_ | _84.4_ | _81.3_ | _85.7_ | _84.5_ | _84.1_ | _83.3_ | _85.8_ | _84.9_ | _75.6_ | _83.4_ | _83.8_ | _84.9_ | _81.7_ | _85.1_ | _84.8_ | _82.7_ | _82.8_ |
| Pediatric vaccination coverage: rubella | _76.5_ | _81.1_ | _78.6_ | _68.9_ | _81.0_ | _76.4_ | _81.3_ | _76.1_ | _82.5_ | _77.5_ | _76.9_ | _82.2_ | _63.1_ | _79.8_ | _79.8_ | _81.1_ | _76.9_ | _81.8_ | _81.8_ | _76.9_ | _80.7_ |
| Dentists | _0.8_ | _0.4_ | _0.6_ | _0.5_ | _0.7_ | _0.7_ | _0.7_ | _0.8_ | _0.6_ | _0.6_ | _0.7_ | _0.6_ | _0.4_ | _0.6_ | _0.6_ | _0.6_ | _0.5_ | _0.7_ | _0.7_ | _0.4_ | _0.6_ |
| Diabetes (age-standardized rates) | _6.7_ | _7.6_ | _9.1_ | _8.4_ | _5.3_ | _4.9_ | _6.5_ | _4.8_ | _5.5_ | _5.2_ | _6.5_ | _5.3_ | _4.0_ | _4.7_ | _7.3_ | _6.0_ | _7.6_ | _5.5_ | _6.1_ | _4.6_ | _5.3_ |
| Demand for contraception satisfied with modern methods in the past 12 months | _11.4_ | _10.8_ | _12.0_ | _12.2_ | _14.2_ | _14.2_ | _13.8_ | _15.5_ | _14.9_ | _12.9_ | _11.7_ | _15.0_ | _15.6_ | _15.3_ | _11.7_ | _14.8_ | _13.2_ | _14.3_ | _13.7_ | _13.5_ | _14.6_ |
| Overweight (age-standardized rates) | _48.1_ | _51.2_ | _49.3_ | _52.5_ | _45.1_ | _43.1_ | _43.2_ | _40.4_ | _41.3_ | _43.5_ | _50.4_ | _40.6_ | _40.0_ | _38.9_ | _50.4_ | _41.3_ | _49.9_ | _42.4_ | _45.0_ | _41.2_ | _43.6_ |
| Pharmacists | _1.1_ | _1.1_ | _1.2_ | _0.7_ | _1.0_ | _1.2_ | _0.8_ | _1.0_ | _0.7_ | _1.2_ | _1.1_ | _0.9_ | _0.7_ | _0.9_ | _0.8_ | _1.2_ | _0.8_ | _1.0_ | _1.2_ | _1.1_ | _1.0_ |
| Smoking (age-standardized rates) | _20.1_ | _19.7_ | _16.8_ | _21.2_ | _20.0_ | _18.7_ | _21.6_ | _21.0_ | _20.0_ | _19.6_ | _19.9_ | _20.8_ | _18.7_ | _16.6_ | _17.8_ | _19.5_ | _20.5_ | _21.2_ | _22.1_ | _18.2_ | _17.4_ |
| Incidence of HIV infections per 100,000 residents (by region of residence) | _3.9_ | _2.0_ | _1.2_ | _3.4_ | _6.3_ | _3.1_ | _7.8_ | _5.8_ | _7.1_ | _4.9_ | _4.0_ | _5.3_ | _2.6_ | _4.7_ | _3.9_ | _3.4_ | _4.5_ | _6.5_ | _5.8_ | _4.7_ | _4.6_ |
| Nurses and midwives | _5.6_ | _5.8_ | _4.4_ | _4.0_ | _5.5_ | _5.9_ | _4.9_ | _6.3_ | _4.6_ | _5.3_ | _6.4_ | _5.1_ | _6.8_ | _6.3_ | _5.1_ | _4.7_ | _4.6_ | _5.5_ | _5.8_ | _5.8_ | _5.4_ |
| Arterial hypertension (age-standardized rates) | _20.3_ | _20.4_ | _23.3_ | _23.1_ | _18.5_ | _16.9_ | _19.7_ | _18.8_ | _18.9_ | _17.2_ | _19.1_ | _18.6_ | _15.4_ | _16.3_ | _21.0_ | _18.9_ | _22.2_ | _16.3_ | _21.6_ | _16.4_ | _18.6_ |
| Physicians | _4.2_ | _3.5_ | _3.9_ | _3.8_ | _4.3_ | _4.1_ | _4.7_ | _3.9_ | _3.3_ | _3.7_ | _4.0_ | _3.6_ | _3.2_ | _3.3_ | _3.7_ | _4.8_ | _4.3_ | _4.3_ | _4.4_ | _4.2_ | _3.8_ |
| Number of deaths in road accidents | _75.7_ | _36.0_ | _102.8_ | _227.6_ | _328.1_ | _74.4_ | _343.2_ | _79.1_ | _445.3_ | _92.1_ | _22.9_ | _249.7_ | _34.7_ | _30.8_ | _223.6_ | _99.2_ | _210.9_ | _232.4_ | _49.9_ | _7.2_ | _315.1_ |
| Percentage of births with more than 4 prenatal check-ups | _78.7_ | _78.2_ | _70.4_ | _76.3_ | _81.2_ | _75.0_ | _-1.0_ | _81.0_ | _78.4_ | _81.6_ | _82.9_ | _79.8_ | _81.4_ | _73.6_ | _76.3_ | _81.5_ | _74.5_ | _78.7_ | _83.2_ | _85.6_ | _76.8_ |
| Day-hospital beds in public and private healthcare institutions | _2.4_ | _3.0_ | _3.2_ | _3.3_ | _1.7_ | _3.1_ | _3.0_ | _3.1_ | _1.7_ | _2.9_ | _3.4_ | _3.3_ | _2.6_ | _3.0_ | _1.5_ | _3.1_ | _2.9_ | _3.1_ | _3.2_ | _2.4_ | _2.2_ |
| Ordinary inpatient beds in public and private healthcare institutions | _23.2_ | _22.7_ | _19.3_ | _20.2_ | _28.4_ | _24.9_ | _24.4_ | _24.9_ | _27.0_ | _24.1_ | _25.5_ | _26.4_ | _26.9_ | _26.9_ | _22.4_ | _23.6_ | _21.6_ | _21.5_ | _24.5_ | _28.0_ | _25.2_ |
| Beds in residential social-welfare and social-healthcare facilities | _38.7_ | _53.2_ | _31.7_ | _15.7_ | _84.0_ | _94.5_ | _36.5_ | _98.1_ | _73.9_ | _68.9_ | _55.4_ | _101.2_ | _104.3_ | _121.5_ | _30.8_ | _45.6_ | _44.4_ | _52.8_ | _51.4_ | _98.1_ | _75.7_ |
| Probability of dying between ages 30 and 69 from cancer, diabetes, cardiovascular, and respiratory diseases | _8.0_ | _8.1_ | _8.6_ | _10.3_ | _7.5_ | _8.0_ | _8.7_ | _8.1_ | _7.8_ | _7.2_ | _8.4_ | _8.4_ | _7.2_ | _6.8_ | _7.9_ | _8.6_ | _9.1_ | _7.6_ | _7.4_ | _8.1_ | _7.3_ |
| Under-five mortality rate | _3.6_ | _4.4_ | _4.7_ | _4.0_ | _3.3_ | _3.3_ | _3.5_ | _3.3_ | _3.3_ | _2.8_ | _3.3_ | _3.0_ | _3.3_ | _3.3_ | _3.6_ | _3.3_ | _4.7_ | _2.8_ | _3.2_ | _4.1_ | _3.1_ |
| Age-specific fertility rate per 1,000 women aged 10–14 | _0.0_ | _0.1_ | _0.1_ | _0.0_ | _0.0_ | _0.0_ | _0.0_ | _0.0_ | _0.0_ | _0.0_ | _0.0_ | _0.0_ | _0.1_ | _0.0_ | _0.1_ | _0.1_ | _0.1_ | _0.0_ | _0.0_ | _0.0_ | _0.0_ |
| Age-specific fertility rate per 1,000 women aged 15–19 | _19.4_ | _16.3_ | _24.9_ | _33.5_ | _19.7_ | _15.9_ | _19.1_ | _23.2_ | _19.6_ | _16.6_ | _16.2_ | _18.7_ | _19.6_ | _16.0_ | _30.3_ | _19.8_ | _48.4_ | _18.2_ | _19.3_ | _14.4_ | _15.6_ |
| Healthy life expectancy at birth | _58.5_ | _55.1_ | _51.9_ | _56.7_ | _60.7_ | _61.1_ | _58.7_ | _60.2_ | _59.8_ | _59.3_ | _57.6_ | _59.0_ | _68.9_ | _64.1_ | _57.6_ | _55.3_ | _56.6_ | _60.8_ | _59.6_ | _60.7_ | _59.6_ |
| Rate of serious injury in road accidents | _34.8_ | _20.5_ | _26.1_ | _22.1_ | _34.0_ | _28.1_ | _36.9_ | _27.9_ | _20.9_ | _27.2_ | _21.4_ | _20.6_ | _32.1_ | _35.3_ | _27.4_ | _32.9_ | _21.8_ | _26.5_ | _21.9_ | _27.3_ | _23.8_ |
| Neonatal mortality rate | _1.8_ | _2.2_ | _2.9_ | _2.1_ | _1.4_ | _1.3_ | _1.8_ | _1.6_ | _1.4_ | _1.0_ | _1.6_ | _1.3_ | _1.6_ | _1.7_ | _1.7_ | _1.5_ | _2.4_ | _1.2_ | _1.5_ | _1.8_ | _1.3_ |
| Mortality rate from road accidents | _5.5_ | _6.1_ | _5.1_ | _3.9_ | _7.0_ | _5.9_ | _5.8_ | _4.8_ | _4.4_ | _5.6_ | _7.1_ | _5.4_ | _6.5_ | _5.6_ | _5.4_ | _5.9_ | _4.1_ | _5.8_ | _5.3_ | _5.8_ | _6.3_ |
| Age-standardized mortality rate from accidental poisoning | _0.3_ | _0.1_ | _0.2_ | _0.1_ | _0.3_ | _0.3_ | _0.6_ | _0.3_ | _0.2_ | _0.6_ | _0.2_ | _0.4_ | _0.1_ | _0.1_ | _0.2_ | _0.5_ | _0.1_ | _0.3_ | _0.2_ | _0.4_ | _0.3_ |
| Age-standardized mortality rate from suicide | _5.5_ | _5.7_ | _4.1_ | _3.0_ | _6.7_ | _7.4_ | _4.1_ | _3.7_ | _5.4_ | _6.5_ | _4.4_ | _6.8_ | _8.1_ | _7.3_ | _4.1_ | _8.0_ | _4.5_ | _5.9_ | _6.9_ | _10.6_ | _6.0_ |
