# Supplementary material for: Unveiling and understanding health inequalities: A bi-clustering study on SDG3 implementation in the Italian regions
Source: PLoS One. 2026 Mar 26;21(3):e0340438. doi: 10.1371/journal.pone.0340438 (PMC13020981; doi:10.1371/journal.pone.0340438)
Supplement: S2 Table — (DOCX) [file pone.0340438.s002.docx]

**S2 Table. Comparison of K-Means vs Bi-clustering.**

| **cluster_id** | **members** | **mean** | **Standard deviation** | **method** |
| --- | --- | --- | --- | --- |
| **0** | Abruzzo; Emilia-Romagna; Friuli-Venezia Giulia; Lazio; Liguria; Lombardia; Marche; Molise; Piemonte; Sardegna; Toscana; Umbria; Veneto | 16.22 | 23.97 | bi-clustering |
| **1** | Basilicata; Calabria; Campania; Puglia; Sicilia | 30.75 | 45.71 |  |
| **2** | Provincia Autonoma di Bolzano/Bozen; Provincia Autonoma di Trento; Valle d'Aosta/Vallée d'Aoste | 44.73 | 40.84 |  |
|  |  | **AVG = 30.57** | **AVG = 36.84** |  |
| **0** | Abruzzo; Basilicata; Calabria; Campania; Lazio; Puglia; Sicilia | 26.14 | 28.20 | kmeans-clustering |
| **1** | Emilia-Romagna; Friuli-Venezia Giulia; Liguria; Lombardia; Marche; Molise; Piemonte; Provincia Autonoma di Trento; Sardegna; Toscana; Umbria; Valle d'Aosta/Vallée d'Aoste; Veneto | 25.17 | 27.86 |  |
| **2** | Provincia Autonoma di Bolzano/Bozen | 26.38 | 29.85 |  |
|  |  | **AVG = 25.9** | **AVG=28.6** |  |
