# Supplementary material for: Unveiling and understanding health inequalities: A bi-clustering study on SDG3 implementation in the Italian regions
Source: PLoS One. 2026 Mar 26;21(3):e0340438. doi: 10.1371/journal.pone.0340438 (PMC13020981; doi:10.1371/journal.pone.0340438)
Supplement: S3 Table — (DOCX) [file pone.0340438.s003.docx]

**S3 Table. Kruskall-Wallis test on cluster independence.**

| **Clusters** | **0** | **1** | **2** |
| --- | --- | --- | --- |
| **0** | 1.000 | **0.004*** | **0.020*** |
| **1** | **0.004*** | 1.000 | 0.185 |
| **2** | **0.020*** | 0.185 | 1.000 |
| **Bi-Clustering** | |  |  |
|  |  |  |  |
| **Clusters** | **0** | **1** | **2** |
| **0** | 1.000 | 0.937 | 0.927 |
| **1** | 0.937 | 1.000 | 0.900 |
| **2** | 0.927 | 0.900 | 1.000 |
| **K-Means clustering** | |  |  |

***Note: With “*” we indicate the independent clusters (i.e. in the bi-clustering cluster 0 is independent from cluster 1 and cluster 2 given its p-value < 0.05. We reject the null hypothesis H_o_: the mean of the groups(clusters) is the same)***
