# Supplementary material for: Unveiling and understanding health inequalities: A bi-clustering study on SDG3 implementation in the Italian regions
Source: PLoS One. 2026 Mar 26;21(3):e0340438. doi: 10.1371/journal.pone.0340438 (PMC13020981; doi:10.1371/journal.pone.0340438)
Supplement: S4 Table — (DOCX) [file pone.0340438.s004.docx]

**S4 Table. ANOVA test on cluster independence for bi-clustering groups**

| **biclusters** | **p-value** | **F-statistics** | **Lower CI** | **Upper CI** |
| --- | --- | --- | --- | --- |
| (0, 1) | 0.007*** | 7.371 | 0.140 | 8.257 |
| (0, 2) | 0.000*** | 20.016 | 0.381 | 22.424 |
| (1, 2) | 0.192 | 1.726 | 0.033 | 1.933 |

***Note: The test is symmetric, meaning the comparison between cluster (0, 2) is equivalent to the comparison between cluster (2, 0)
H_o_: the mean of the three groups(clusters) is the same. *, **, and *** indicate significance at the 10 percent, 5 percent, and 1 percent levels, respectively. We reject the null hypothesis; this suggests that there are significant differences for (0,1) and (0,2) clusters.***
