# Supplementary material for: Unveiling and understanding health inequalities: A bi-clustering study on SDG3 implementation in the Italian regions
Source: PLoS One. 2026 Mar 26;21(3):e0340438. doi: 10.1371/journal.pone.0340438 (PMC13020981; doi:10.1371/journal.pone.0340438)
Supplement: S5 Table — (DOCX) [file pone.0340438.s005.docx]

**S5 Table. ANOVA test on cluster independence for k-means groups**

| **clusters** | **p-value** | **F-statistics** | **Lower CI** | **Upper CI** |
| --- | --- | --- | --- | --- |
| (0, 1) | 0.816 | 0.054 | 0.001 | 0.055 |
| (0, 2) | 0.953 | 0.003 | 0.000 | 0.004 |
| (1, 2) | 0.646 | 0.212 | 0.004 | 0.215 |

***Note: The test is symmetric, meaning the comparison between cluster (0, 2) is equivalent to the comparison between cluster (2, 0)
H_o_: the mean of the three groups(clusters) is the same. *, **, and *** indicate significance at the 10 percent, 5 percent, and 1 percent levels, respectively.*** ***ANOVA test works by comparing the full groups. We accept Ho, this means that the clusters are the same, and there are no significant differences between the clusters.***
