# Supplementary material for: Unveiling and understanding health inequalities: A bi-clustering study on SDG3 implementation in the Italian regions
Source: PLoS One. 2026 Mar 26;21(3):e0340438. doi: 10.1371/journal.pone.0340438 (PMC13020981; doi:10.1371/journal.pone.0340438)
Supplement: S6 Table — (DOCX) [file pone.0340438.s006.docx]

**S6 Table. Tukey-HSD test for independent groups for bi-clustering**

| **bi-clusters** | **Tukey-HSD Statistic** | **p-value** | **Lower CI** | **Upper CI** |
| --- | --- | --- | --- | --- |
| (0 ,1) | -14.527 | 0.024** | -27.497 | -1.556 |
| (0 ,2) | -28.511 | 0.001*** | -47.510 | -9.512 |
| (1, 0) | 14.527 | 0.024** | 1.556 | 27.497 |
| (1,2) | -13.984 | 0.222 | -33.830 | 5.861 |
| (2, 0) | 28.511 | 0.001*** | 9.512 | 47.510 |
| (2 ,1) | 13.984 | 0.222 | -5.861 | 33.830 |

***Note:*** ****, **, and *** indicate significance at the 10 percent, 5 percent, and 1 percent levels, respectively. H_o_: the mean of the two groups(clusters) is the same.*** ***Tukey test works by comparing pairs and not the full groups. We reject the null hypothesis; this suggests that there are significant differences for the pairs (0,1) and (0,2) (0,1) and (2,0) clusters.***
