# Supplementary material for: Unveiling and understanding health inequalities: A bi-clustering study on SDG3 implementation in the Italian regions
Source: PLoS One. 2026 Mar 26;21(3):e0340438. doi: 10.1371/journal.pone.0340438 (PMC13020981; doi:10.1371/journal.pone.0340438)
Supplement: S7 Table — (DOCX) [file pone.0340438.s007.docx]

**S7 Table. Tukey-HSD test for independent groups for k-Means**

| **clusters** | **Tukey-HSD Statistic** | **p-value** | **Lower CI** | **Upper CI** |
| --- | --- | --- | --- | --- |
| (0 ,1) | 0.972 | 0.929 | -5.289 | 7.234 |
| (0 ,2) | -0.242 | 0.999 | -12.765 | 12.281 |
| (1, 0) | -0.972 | 0.929 | -7.234 | 5.289 |
| (1,2) | -1.215 | 0.975 | -14.498 | 12.068 |
| (2, 0) | 0.242 | 0.999 | -12.281 | 12.765 |
| (2 ,1) | 1.215 | 0.975 | -12.068 | 14.498 |

***Note: We reject the null hypothesis H_o_: the mean of the two groups(clusters) is the same.***
