# Supplementary material for: Unveiling and understanding health inequalities: A bi-clustering study on SDG3 implementation in the Italian regions
Source: PLoS One. 2026 Mar 26;21(3):e0340438. doi: 10.1371/journal.pone.0340438 (PMC13020981; doi:10.1371/journal.pone.0340438)
Supplement: S8 Table — (DOCX) [file pone.0340438.s008.docx]

**S8 Table. Cohen’s d effect size for bi-clustering and k-means**

| **clusters** | **biclust** | **k-means** |
| --- | --- | --- |
| (0,1) | 0.395 | 0.035 |
| (0,2) | 0.837 | 0.008 |
| (1,2) | 0.318 | 0.042 |

***Note: The Cohen’s d coefficient is a standard measure of effect size that quantifies the magnitude of the difference between the means of two groups. The measure is conventionally interpreted as follows:0 - 0.2: Small effect size (negligible difference).0.2 - 0.8: Medium effect size.> 0.8: Large effect size.*** ***Based on this metric, the bi-clustering results yielded stronger distinctions: all bi-clusters demonstrated at least a medium effect size, with clusters 0 and 2 achieving a large effect size. Conversely, all k-Means clusters showed a small effect size, indicating that their group averages were too similar to demonstrate a meaningful difference.***
