# Supplementary material for: Unveiling and understanding health inequalities: A bi-clustering study on SDG3 implementation in the Italian regions
Source: PLoS One. 2026 Mar 26;21(3):e0340438. doi: 10.1371/journal.pone.0340438 (PMC13020981; doi:10.1371/journal.pone.0340438)
Supplement: Attachment — Submitted filename: Response to reviewers.docx [file pone.0340438.s014.docx]

**Authors’ response to Reviewers’ comments**

**PONE-D-25-29410**

**Unveiling and Understanding Health Inequalities: A Bi-Clustering Study on SDG3 Implementation in the Italian regions**

The authors of this paper would like to thank the Editor and reviewers of PlosOne for their insightful comments and constructive suggestions, which have helped us to significantly improve the quality and clarity of our manuscript.

Please note that the revised manuscript has been submitted in Track Changes mode, allowing you to easily identify all modifications made in response to the reviewers’ feedback. Additionally, to facilitate the review process, each change in the text is accompanied by a comment indicating which specific reviewer comment it addresses.

| N | Editor’s comments | Authors’ responses |
| --- | --- | --- |
| 1.a | Be sure to:  Align the main objective of study (SDG3 Strategies) with results and discussion section. | We thank the Editor for this suggestion.  In response to this comment, as well as to the reviewers’ suggestions, the paper has been strongly integrated and revised. Beginning from the abstract, we carefully restructured and refined the manuscript to ensure that the main objective of the study is consistently aligned with the results and discussion sections. We hope that these revisions have now better focused the study and strengthened the coherence between the research aims, findings, and their interpretation. See changes in sections:  **- *Abstract,***  **- 1 - *Introduction,***  **- 3.2 (Results) - *Analysis of SDG3 trends across identified cluster,***  **- 3.2.1 (Results) - *Intra-cluster similarities and inter-cluster divergences: the role of infrastructure variables and cost containment policies***, and  **- 4 - *Discussion.*** |
| 1.b | Provide detailed practical insights and recommendations on SDG3 implementation in the Italian region. | Following this suggestion, we improved the conclusion section as follows:  *“Our findings reveal how a decentralized health system, coupled with austerity measures and socio-economic disparities, has led to divergent health outcomes across Italy. We show that a one-size-fits-all approach is ineffective and that targeted governance measures are essential for each region. Policymakers should direct greater investment toward initiatives that advance SDG3, especially in underserved and economically disadvantaged regions, optimizing resource allocation to improve health service management where the need is most urgent. We also highlight the adverse effects of the RRPs, which, while reducing regional debt, inadvertently worsened healthcare indicators”*.  (See section **5 -** **Conclusion)** |
| 1.c | Develop coherence from introduction to the conclusion and align each section. | We would like to thank the Editor for this valuable suggestion. The entire paper has been substantially revised in line with the comments from both the Editor and the reviewers, with the specific goal of developing coherence from the introduction to the conclusion and ensuring a clear alignment across all sections. |
| **N** | **Reviewer 3’s comments** | **Authors’ responses** |
| 1 | The abstract does not comprehensively reflect the full content of the manuscript and requires revision. It is recommended that the term “Sustainable Development Goals” be written out in full the first time it appears in the abstract, followed by its abbreviation (SDGs). | Point taken. We have corrected the term “Sustainable Development Goals” and revised the abstract as follows:  “*Health equity is a key policy priority in public health and a central component of Sustainable Development Goal 3 (SDG 3), which focuses on “Good Health and Well-Being.” While SDG 3 sets global targets, its success depends on the capacity of local actors to translate these objectives into effective regional actions. This constitutes a major challenge for countries with decentralized healthcare systems and marked territorial disparities, as in the case of Italy.*  *Implementing SDG 3 targets at the national level requires adapting strategies to diverse regional contexts in order to promote and sustain healthy lives and well-being across the population. In this regard, there is a critical need for research that explores differences among regional healthcare systems to understand how global goals can be translated into meaningful societal outcomes. By monitoring health outcomes at the regional level, policymakers can identify barriers to SDG 3 implementation and promote more effective, equity-oriented policies.*  *This study addresses this gap by adopting a subnational perspective that responds to calls for moving beyond national averages and identifying context-specific barriers and enablers of SDG 3. Using spectral bi-clustering, an innovative data mining technique, we examine whether some SDG 3 indicators are more relevant across different clusters of Italian regions, thereby uncovering the leading factors of SDG 3 performance and potential obstacles to achieving these targets. Our findings provide actionable policy guidance, showing that effective implementation of SDG3 in Italy requires regionally tailored strategies rather than a one-size-fits-all approach. By identifying the specific health system gaps and strengths of each cluster of regions, we offer evidence to support more equitable and targeted policy interventions . This study is among the first to apply spectral bi-clustering to a regional health equity analysis*” (See the **Abstract).** |
| 2 | The introduction and background sections should be merged and summarized, in accordance with the journal’s formatting guidelines. | We have merged and streamlined the former “Introduction” and “Background” sections into a single section now entitled *“Introduction”*. In addition, we have removed Table 1. Instead, we now provide a footnote in the *Introduction* that contains the link to the official document with the full description of targets and indicators for all SDGs. Finally, in Table 2 we have added a new column titled *“Unit of measure”*. (See section **1 -** **Introduction).** |
| 3 | In the Data section, please specify the variables included in the ISTAT database, along with their characteristics and measurement scales | The data included in our study, along with their characteristics and measurement scale, are presented in Table S1 of the **supplementary materials**. For greater clarity, we have revised the section to explicitly draw attention to the supplementary materials, as follows:  “*The list of indicators for Italy is detailed in Table 1, and with full database in Supplementary Material (Table S1)*” (See section **2.2 –** **Data).** |
| 4 | Some of the content currently under the “Clustering Model” subsection in the Results should be moved to the Methods section, where it is more appropriate. | Based on your request, we have moved some of the content currently under the “Clustering Model” subsection in the Results section to the Methods section, as it is more appropriate there.  “...*Specifically, we began the analysis by making an average of the SDG3 indicators from the ISTAT database over the 2013-2019 period. For each Italian region, we computed a single value for every SDG3 variable by averaging the indicator values over these years (see Supporting Information - S1 Table). Using this dataset, we applied a bi-clustering algorithm to identify clusters of Italian regions. The algorithm requires a pre-specified number of clusters to be formed. Starting from the work of Gandullia and Leporatti, we considered three homogeneous groups, which align with the structural and organizational differences of the Italian Regional Health Systems (RHSs) and their socio-economic characteristics. This distinction is based on factors such as per capita healthcare expenditure, managerial efficiency, and the quality of services, suggesting a natural segmentation into northern, central, and southern macro-areas. Accordingly, we imposed the number of clusters K = 3 to identify which SDG3 variables are most influential within each regional cluster. We applied the SpectralBiClustering algorithm on the data from S1 Table to achieve this*” (See section **2.1 – Bi-clustering technique).** |
| 5 | The rationale for selecting each indicator for each cluster, as well as the selection method, should be clearly explained in the Methods section | Thank you to the reviewer for this interesting suggestion. We’ve revised the manuscript to incorporate your feedback as follows:  *“....It is important to note that Bi-Clustering is a fully unsupervised method, that after the indication of “number of clusters” has no need of further hyper-parameters. The method itself extracts the most relevant indicators to each bi-cluster (in our case clustering geographical regions, and health indicators at the same time). We can further study individual health indicators and their relevance per cluster by comparing each cluster health indicator with its national average…..”.* (See section **2.1- Bi-Clustering technique**). |
| 6 | The Discussion section is not well-developed. It primarily reiterates the results without offering sufficient interpretation, comparison with other studies, or practical implications for the key findings. | The authors would like to thank the reviewer for this valuable comment. In response, the Discussion section has been strongly expanded to incorporate a deeper interpretation of the results as well as the practical implications of the key findings (See **section 4 – Discussion**). With regard to the comparison with other studies, we clarified—particularly in the Introduction section, as suggested by another reviewer—the lack of similar research. Specifically, *while cross-country comparisons highlight Italy’s national performance, no studies to date have examined how specific SDG 3 targets are differentially expressed across Italian regions*. Nevertheless, in line with another reviewer’s suggestion, the Introduction now also includes a brief reference to how SDG 3 has been addressed in other countries, in order to situate Italy’s case within a broader global context (See section **1 – Introduction**). |
| 7 | The Conclusion section is overly long. It should be shortened and focused on drawing conclusions directly based on the specific findings of this study. | Thank you for this helpful suggestion. We have revised the Conclusion section to better focus on the specific findings of our study, as recommended. However, in response to the other requests, we also integrated a discussion of the study’s limitations and recommendations for future research within the same section. For this reason, despite our efforts to streamline the text, the overall length of the Conclusion could not be reduced (See section **5 – Conclusion**). |
| 8 | The study’s limitations should be clearly stated, along with recommendations for future research. | We have included the study’s limitation and the recommendations for future research in the conclusion (See section **5 – Conclusion**). |
| 9 | A particularly important point is that the main aim of the study is to propose strategies for translating global and national policies and goals into localized interventions and actions, taking into account regional economic, social, and cultural differences. However, this core objective is scarcely addressed in the Results and Discussion sections and should be emphasized more clearly. | We thank the reviewer for this important observation. We would like to clarify that the primary aim of our study is not to propose strategies for translating global and national policies and goals into localized interventions and actions, but rather to contribute to this process by providing robust tools for monitoring health outcomes at the regional level. *Our work responds to the critical need for research that analyzes differences among regional healthcare systems to better understand how global objectives, such as SDG 3, can be effectively translated into meaningful societal change.* *By applying a bi-clustering approach to SDG3 indicators, our analysis highlights regional disparities and identifies context-specific health system barriers.* In this way, our study provides policymakers with actionable evidence on where implementation gaps exist, and which areas require targeted investment or policy reform. Monitoring health outcomes at the regional level is thus a fundamental step to informing them about the design of effective, equity-oriented policies. We have revised the manuscript to emphasize this contribution more clearly in the Abstract, Results and Discussion sections, underlining that our findings serve as a diagnostic tool for identifying regional divergences in SDG3 implementation rather than as prescriptive strategies. (See sections: **Abstract***;* **3.2 -** Analysis of SDG3 trends across identified cluster (Results); **4** **-** Discussion). |
| **N** | **Reviewer’s comments (attached file)** | **Authors’ responses** |
| 1 | A) Methodology  The use of Spectral Bi-Clustering enables multidimensional analysis and allows for simultaneous clustering of both regions and indicators—an advantage over traditional one-dimensional clustering.  Recommendations for improvement:  • The methodological section would benefit from more technical detail, such as how normalization, variance filtering, or missing data handling were conducted.  • The advantages of bi-clustering over other methods (e.g., k-means, PCA) should be demonstrated with a concrete example.  • Details on software or libraries used (e.g., Python, R, Scikit-learn) should be specified. | We thank the reviewer for the suggestion. In response to this comment, we’ve added a new part in the method sub-section (Bi-clustering technique):  “....*To maintain the integrity of our unsupervised approach, data pre-processing was kept to a minimum. We used data from 2013 to 2023 but excluding COVID-19 years (2020-2023). We treated this seven-year period as “homogeneous” by calculating the average value per indicator for each region. This approach ensured that our final dataset contained no missing values and retained the original unit of measure for each variable.*  *Before clustering, a z-score transformation (centering the mean and scaling by the standard deviation) was applied to the data. This crucial step ensured that all variables were on the same scale, which is a prerequisite for reliable clustering and allows the algorithm to form meaningful bi-clusters of both indicators and regions.*  *We compared the performance of biclustering against a traditional method, K-Means, using two key tests. We used K-Means and we applied the same number of clusters to our biclustering model (Supplementary Materials Table S2).*  *The first test evaluated each method’s ability to identify meaningful groups of regions based on their socio-economic characteristics and statistical power. Since our data and cluster groups did not meet the assumptions of a normal distribution or equal variance, we used the non-parametric Kruskal-Wallis H-test instead of the traditional ANOVA f-test to assess for significant differences between cluster means. The results of these tests, detailed in the supplementary materials (Table S2, S3), provide a clear comparison:*  *-K-Means divides regions based on all health indicators, creating groups with similar means and standard deviations. In fact, the algorithm works by minimizing the within-cluster variance (inertia) by finding the centroids of data points using Euclidean distances.  - Bi-Clustering, instead, by allowing for partial expression of variables, is able to separate not only regions (rows), but also health indicators (columns) making it feasible to create more independent clusters where independence can be seen (at least partially) with ANOVA like tests. (Kruskal-Wallis test for independence of two populations is used).*  *In summary, statistical tests show that Bi-clustering builds better separation, and (partial) cluster independence (in the Table S3 in the Supplementary Materials cluster 0 is different from 1 and 2, but cluster 1 is not independent from cluster 2).  Conversely, K-Means tends to rearrange members in order to have a smaller within - cluster variance (inertia) and a general smaller in-cluster variance renouncing to have independent clusters.*  *While statistical tests provide valuable insights, we believe a more complete understanding comes from evaluating the results against real-world* ***s****ocio-economic conditions in Italy. The traditional division of Italy into North, Center, and South, combined with knowledge of regional healthcare systems, strongly supports the partitions created by biclustering. For example, biclustering grouped Valle d’Aosta, Trento, and Bolzano/Bozen together, which is a more accurate reflection of their similar socio-economic history than the K-Means result, which only clustered Bolzano/Bozen.*  *In conclusion, our comparison with K-Means demonstrates two significant advantages of using biclustering for this dataset: a) It produces clusters with greater statistical separation and partial independence due to its ability to use only a subset of relevant variables.; b) It accurately reflects the socio-economic geography of Italy’s regions, producing more meaningful and interpretable clusters.*  *The analysis was performed using the Python Scikit-learn library, specifically the SpectractBiClustering and K-Means functions”.*  (See section **2.1 -** **Bi-clustering technique)** |
| 2 | B) Abstract - Suggestions:  B.1 - The final sentences should explicitly state the study’s contribution to the field, such as: “This study is among the first to apply spectral bi-clustering in regional health equity analysis.”  B.2 - A short sentence on the policy implications would strengthen its relevance and appeal. | Following these suggestions, we integrated the abstract as follows:  “….*Our findings provide actionable policy guidance, showing that effective implementation of SDG3 in Italy requires regionally tailored strategies rather than a one-size-fits-all approach. By identifying the specific health system gaps and strengths of each cluster of regions, we offer evidence to support more equitable and targeted policy interventions. This study is among the first to apply spectral bi-clustering to a regional health equity analysis*”. (See the **Abstract).** |
| 3 | C) Introduction - Suggestions:  C.1 - The research gap should be emphasized more explicitly. | According to this suggestion, we integrated the paper as follows:  “…..*Starting from this context, this paper is focused on the Italian case, a country in which the regional healthcare systems show a high degree of autonomy and can be seen as individual cases within the nation. While cross-country comparisons situate Italy’s national performance, no studies have investigated how specific SDG 3 targets are differentially expressed across Italian regions.* *Our research addresses this gap by offering a subnational perspective that responds to calls for moving beyond averages and identifying contextualized barriers and enablers of SDG 3 implementation….*” (See section **1 – Introduction**). |
|  | C.2 - The significance of the research questions could be elaborated—why are they important for policymakers or the SDG agenda? | Thank you for your feedback. We agree that a more detailed explanation of the research questions’ significance is essential to underscore the paper’s contribution. Accordingly, we integrated “**Introduction**” section as follows:  “…*.*Using an innovative data mining technique called bi-clustering, this study seeks to address two key research questions RQs) that have significant implications for policymakers and the broader SDG agenda:  *1. “Which of the SDG3 variables are more expressed within a given territory (regions), and how Italian regions can be grouped to yield meaningful insights into local health-related trends?” ……...* ***This capability is crucial for policymakers because it helps to pinpoint the specific, localized drivers of health outcomes that might otherwise be obscured by national or regional averages***…….  *2. Which variables within each cluster are most distant from the national average?” ……* ***This enables policymakers to create truly “tailored” interventions aimed at achieving SDG3 targets, moving beyond a one-size-fits-all approach and promoting genuine health equity***….” |
| 4 | D) Literature Review / Background - Suggestions:  D.1 - A clearer comparison with existing bi-clustering applications in public health is needed to justify the choice of method. | We sincerely thank the Reviewer for this valuable observation.  To the best of our knowledge, no studies to date have applied bi-clustering techniques specifically within the field of public health. Existing applications are largely concentrated in computational science and bioinformatics, where bi-clustering has been used for gene expression analysis and related problems (e.g., Kluger et al., 2003; Castanho et al., 2024). In economics, a few studies have extended the method to analyze the interplay between industrial sectors and regional economies (e.g., Chao et al., 2021; Haedo, 2022). However, despite its strong potential to capture complex, multidimensional relationships, bi-clustering has not yet been employed to investigate public health disparities. This absence of precedent is precisely the main strength of our study: by introducing bi-clustering to the analysis of health inequalities and Sustainable Development Goal 3 (SDG3), we expand the methodological toolkit available for public health research. The technique allows us to uncover “partial expressions” of health indicators across regions, a feature that traditional clustering methods or panel analyses cannot capture as effectively. We believe this novel application significantly enhances the capacity to identify hidden patterns and region-specific priorities, offering policymakers evidence-based insights that go beyond aggregate national averages. |
|  | D.2 - A brief mention of how SDG3 has been addressed in other countries could situate Italy’s case within a broader global context. | According to this suggestion, we integrated the paper as follows:  “…*At the European level, some studies have assessed SDG 3 through cross-national dashboards and inequality monitoring frameworks. Comparative analyses highlighted substantial heterogeneity across Member States, revealing clusters of strengths and persistent gaps often hidden by national averages. Country-specific evidence reinforced these findings: Sweden’s longstanding path toward universal health coverage illustrates how system design shapes SDG 3 delivery, while Spain-focused studies, drawing on GBD indicators and regional well-being metrics, show how resource allocation and risk factors translate into territorial disparities*….” (See section **1 – Introduction**). |
|  | D.3 - The literature gap should be explicitly stated and linked to the research questions. | Thank you for your valuable comment. In response, we have revised the introduction to make the literature gap more explicit and directly connected to the research questions. Specifically, we now state:  “*Starting from this context, this paper is focused on the Italian case, a country in which the regional healthcare systems show a high degree of autonomy and can be seen as individual cases within the nation. While cross-country comparisons situate Italy’s national performance, no studies have investigated how specific SDG 3 targets are differentially expressed across Italian regions. Our research addresses this gap by offering a subnational perspective that responds to calls for moving beyond averages and identifying contextualized barriers and enablers of SDG 3 implementation*”. (See section **1 – Introduction**).  This passage has been placed immediately before the research questions to strengthen their justification and to clearly highlight the gap that our study aims to fill. |
| 5 | E) Methods - Suggestions:  E.1 - Details regarding data pre-processing (e.g., normalization, missing data, outliers) should be added. | Data from 2013-2023 have been limited to the interval 2013-2019 to remove potential biases due to the Covid-19 pandemics. Then, given the 7 years the average along these years has been taken to produce a dataset of regions/health indicators that keeps the average behaviour and removes trends. Clustering is applied to this dataset of regions, and health indicators only after re-scaling each variable with z-score transformation (removing mean and dividing per std). In this way all variables can be used in clustering without the risk of misrepresenting the importance of a single indicator.  However, according to this comment, the paper was integrated as follows:  “*To maintain the integrity of our unsupervised approach, data pre-processing was kept to a minimum. We used data from 2013 to 2023 but excluding COVID-19 years (2020-2023). We treated this seven-year period as “homogeneous” by calculating the average value per indicator for each region. This approach ensured that our final dataset contained no missing values and retained the original unit of measure for each variable*” (See section **2.1 – Bi-clustering technique**). |
|  | E.2 - The statistical significance of cluster differences (e.g., ANOVA, t-tests) is not discussed—this would enhance methodological rigor | According to this comment, we integrated the paragraph by adding a section on the statistical significance of cluster differences with a comparison with the traditional method K-Means. The conclusion is that the bi-clustering method is at least in part able to create independent clusters (different mean and variance within clusters), while, for this dataset, the K-Means methodology creates groups of points (regions) that on their health indicators are completely correlated and non-independent. Also, another reason that justifies the advantage of the Bi-Clustering methodology is its ability, at least in this specific case, to recover some of the socio-economic characteristics of the Italian regions that add up for Northern Italy regions (See the trach-changes in section **2.1 – Bi-clustering technique**). |
|  | E.3 - Software used and parameter settings should be described | According to this comment, we integrated the paper as follows:  “*It is important to note that Bi-Clustering is a fully unsupervised method, that after the indication of “number of clusters” has no need of further hyper-parameters. The method itself extracts the most relevant indicators to each bi-cluster (in our case clustering geographical regions, and health indicators at the same time). We can further study individual health indicators and their relevance per cluster by comparing each cluster health indicator with its national average……*  *….The analysis was performed using the Python Scikit-learn library, specifically the SpectractBiClustering and K-Means functions*.”  (Section **2.1 – Bi-clustering technique).** |
|  | E.4 - A clearer justification for the selection and inclusion/exclusion of SDG3 indicators would be helpful. | The justification for the selection and inclusion/exclusion of SDG3 indicators was integrated as follows:  “*The selection of SDG3 indicators was guided by both conceptual relevance and data availability in the Italian context. First, we included all SDG3 indicators officially reported by ISTAT [63], which provides harmonized regional-level data consistent with the United Nations framework. This ensured that the analysis was grounded in indicators already validated for monitoring progress toward SDG3 at the national level. Second, we excluded indicators* *that are not applicable to Italy, such as those related to tropical diseases (e.g., Malaria and Tuberculosis), since they are not measured or relevant in the Italian epidemiological context. This exclusion avoided introducing variables with no variance across regions, which would have biased the clustering results. Third, we focused on indicators available for a sufficiently long- and homogeneous-time span (2013–2019), excluding years affected by the COVID-19 pandemic (2020–2023) to prevent distortions caused by this exceptional shock*”. (See section **2.2 – Data**) |
| 6 | F) Discussion - Suggestions:  F.1 - Comparison with previous studies is limited. The discussion would be stronger if the findings were contrasted with existing literature. | The authors would like to thank the reviewer for this valuable comment. In response, the Discussion section has been expanded as required according to the other comments of reviewers. With regard to the comparison with other studies, we clarified—particularly in the Introduction section, the lack of similar research. Specifically, *while cross-country comparisons highlight Italy’s national performance, no studies to date have examined how specific SDG 3 targets are differentially expressed across Italian regions*. Nevertheless, in line with another reviewer’s suggestion, the Introduction now also includes a brief reference to how SDG 3 has been addressed in other countries, in order to situate Italy’s case within a broader global context. |
|  | F.2 - The limitations of bi-clustering (e.g., sensitivity to initial conditions, lack of time series integration) should be discussed explicitly. | In response to this comment the paper was integrated as follows:  “....*Despite our promising findings, it’s important to acknowledge some limitations. First, the data for SDG3 targets in Italy only begins in 2013, and the available time series data is short (up to 2023). The COVID-19 pandemic (2020-2023) also severely disrupted previous trends, which forced us to restrict our analysis to the 2013-2019. While this choice was necessary to avoid distortions introduced by the extraordinary impact of the pandemic on health systems and SDG3 indicators (e.g., sudden reductions in hospital capacity, shifts in healthcare priorities, and changes in population health behaviours), it also implies that our results primarily reflect pre-pandemic dynamics. Consequently, the regional disparities identified in this study should be interpreted as representative of structural and long-term features of the Italian healthcare system, rather than of its performance under emergency conditions……*  *Second, our use of spectral bi-clustering has limitations. This method is sensitive to data characteristics and can be unstable. As a “global” method, it may not be effective at identifying clusters that exist at different scales within the same dataset. Furthermore, our application, which evolves time series data, does not inherently account for the sequential nature of time, as it treats all time points (columns) as independent features”* (See section **5 – Conclusion**). |
|  | F.3 - The exclusion of 2020–2021 data due to COVID-19 is justified, but its implications on generalizability should be acknowledged. | According to this comment, section **5 (Conclusion)** was integrated as follows:  “….*While this choice was necessary to avoid distortions introduced by the extraordinary impact of the pandemic on health systems and SDG3 indicators (e.g., sudden reductions in hospital capacity, shifts in healthcare priorities, and changes in population health behaviours), it also implies that our results primarily reflect pre-pandemic dynamics. Consequently, the regional disparities identified in this study should be interpreted as representative of structural and long-term features of the Italian healthcare system, rather than of its performance under emergency conditions. Future research will be needed to assess how the pandemic has reshaped these disparities and whether the clusters we identified have persisted, converged, or diverged in the aftermath of COVID-19*”. |
| 7 | G) Conclusion - Suggestions:  G.1 - The theoretical and methodological contributions of the study should be clearly stated. | According to the reviewer’s suggestion we have rewritten the “**Conclusion**” section as follows:  “*This study makes several key contributions by demonstrating the utility of bi-clustering as a powerful tool for analysing health data in a localized context. While traditional methods like standard clustering or panel data analysis can provide a broad overview, they often fail to capture the specific structural disparities present in heterogeneous systems like Italy’s. Our approach addresses this by identifying “partial expressions” of complex variables, offering a more granular understanding of SDG implementation at the regional level. Our findings reveal how a decentralized health system, coupled with austerity measures and socio-economic disparities, has led to divergent health outcomes across Italy. We show that a one-size-fits-all approach is ineffective and that targeted governance measures are essential for each region…”.* |
|  | G.2 - A short reflection on the utility of bi-clustering for SDG monitoring in other national contexts would add depth. | We thank this reviewer for this suggestion. The paper was integrated as follows:  *“….The methodology we’ve applied in Italy can be a valuable model for monitoring SDGs in other national contexts, particularly those with significant internal disparities. The bi-clustering approach is not limited to SDG3 and can be extended to other indicators. For example, countries with decentralized or devolved governance structures, such as India, Brazil, or the United States, could use this method to identify which specific SDG variables are most critical for their sub-national regions. By identifying these “partial expressions”, policymakers can move beyond national-level averages and design interventions tailored to local needs. This could reveal important regional differences in areas like poverty (SDG1), education (SDG4), or clean water access (SDG6), allowing for more effective and targeted resource allocation and policy development”.* (See section **5 – Conclusion).** |
|  | G.3 - The future research directions section could be expanded—for example, exploring SDG indicators beyond SDG3, or applying this approach in other decentralized health systems. | According to this comment, section **5 (Conclusion)** was integrated as follows:  *“Our study opens several points for future research as: a) Expanded SDG Indicators: future studies could apply the bi-clustering approach to other SDG indicators beyond SDG3 in Italy or other countries with pronounced regional disparities. This could help identify which variables are most critical for driving progress toward the UN’s sustainable development goals in diverse sectors; b) De-centralized Health Systems: the approach can be applied to other countries with decentralized health systems to identify their critical variables and structural disparities. This would allow for cross-country comparisons and the development of best practices for policy implementation…..”.* |
