# Supplementary material for: Unveiling and understanding health inequalities: A bi-clustering study on SDG3 implementation in the Italian regions
Source: PLoS One. 2026 Mar 26;21(3):e0340438. doi: 10.1371/journal.pone.0340438 (PMC13020981; doi:10.1371/journal.pone.0340438)
Supplement: Attachment — Submitted filename: SDG3 Italian Region.docx [file pone.0340438.s013.docx]

**Unveiling and understanding health inequalities: a Bi Clustering study on SDG3 implementation in the Italian regions**

**Reviewer Report – Major Revision**

Dear Authors,

I have carefully reviewed the revised manuscript and your responses to the previous comments. While some aspects have been improved, several important points remain unaddressed or only partially covered. Below I provide a detailed assessment and recommendations.

**Strengths:**

- The manuscript addresses an important global issue by clustering countries using SDG3 indicators.
- Bi-clustering is a relatively novel application in this context and has the potential to reveal meaningful results.
- The revision has added more information on preprocessing (normalization, handling of missing data) and included some new references.

**Weaknesses and Recommendations:**

1. **Methodology**
   - Although details on normalization and missing data were added, the rationale for parameter choices (e.g., variance thresholds, similarity measures, algorithm settings) remains insufficient. These choices should be justified, including their potential effect on robustness.
   - The most critical weakness is that cluster differences are presented only descriptively. To strengthen the validity of the results, they should be supported with **statistical tests**. For example:
     - Use **ANOVA** or **t-tests** to demonstrate whether clusters differ significantly on SDG3 indicators.
     - Apply **post-hoc tests** (e.g., Tukey’s HSD) to identify which clusters differ.
     - Report effect sizes and p-values to establish the meaningfulness of the clusters.
   - Without such validation, it is unclear whether the clusters are statistically distinct or artifacts of the algorithm.
2. **Motivation**
   - The rationale for using bi-clustering remains weak. Please clarify why this method was chosen over conventional techniques (k-means, hierarchical clustering, PCA) and what unique insights it offers in the SDG3 context.
3. **Literature Review**
   - The literature review has been improved but still does not clearly define the research gap.
   - Please explicitly state what prior studies have done, where they fall short, and how your study differs.
   - Include more recent SSCI/SCI-indexed works and international reports, especially those discussing design–outcome relationships, financing barriers, and local variation.
4. **Research Question**
   - The research question is implied but not explicitly stated. Please formulate it clearly (e.g., *“How can bi-clustering of SDG3 indicators reveal distinct groups of countries, and what policy implications follow?”*).
5. **Abstract**
   - The abstract is overly long, repetitive, and contains too many technical details. Please rewrite it more concisely, covering aim, method, key findings, and implications.
6. **Discussion and Policy Implications**
   - The discussion includes very general recommendations (e.g., “countries should cooperate”). Recommendations should be **concrete and measurable**, such as:
     - Indicating which low-performing countries should prioritize specific SDG3 indicators.
     - Suggesting mechanisms for knowledge transfer from high-performing clusters.
     - Explaining how international organizations might use clusters for resource allocation.
7. **Diversity of Recommendations**
   - Current recommendations are one-dimensional. Please tailor them to different stakeholders:
     - **Policymakers:** concrete policy actions for low- and middle-income countries.
     - **Researchers:** directions for extending bi-clustering to other SDGs.
     - **International agencies:** implications for funding and monitoring.
8. **Originality**
   - At present, the claim of originality rests mainly on the novelty of applying bi-clustering in this context.
   - This needs to be strengthened by comparing your results more explicitly with prior clustering studies and by showing how your approach contributes **methodologically or theoretically**, not only through application.

**Conclusion**
The paper addresses a valuable topic but requires substantial revisions before it can be considered for publication. In particular, the **methodology must be statistically validated**, and the **motivation, literature gap, and originality** should be articulated more convincingly. The abstract, discussion, and recommendations also require clearer and more concrete presentation. With these improvements, the manuscript could make a stronger contribution.
