# Supplementary material for: Unveiling and understanding health inequalities: A bi-clustering study on SDG3 implementation in the Italian regions
Source: PLoS One. 2026 Mar 26;21(3):e0340438. doi: 10.1371/journal.pone.0340438 (PMC13020981; doi:10.1371/journal.pone.0340438)
Supplement: Attachment — Submitted filename: Response to Reviewers R2.docx [file pone.0340438.s015.docx]

**Authors’ response to Reviewers’ comments**

**PONE-D-25-29410R1**

**Unveiling and Understanding Health Inequalities: A Bi-Clustering Study on SDG3 Implementation in the Italian regions**

The authors of this paper would like to thank the reviewers of PLOS ONE for their insightful comments and constructive suggestions, which have helped us significantly improve the quality and clarity of our manuscript. Please note that the revised manuscript has been submitted in Track Changes mode (Revised Manuscript with Track Changes), allowing you to easily identify all modifications made in response to the reviewers’ feedback. Additionally, a clean version of the manuscript has been submitted under the filename “Manuscript”.

|  | **Reviewer’s comments** | **Authors’ responses** |
| --- | --- | --- |
| **Methodology** | Although details on normalization and missing data were added, the rationale for parameter choices (e.g., variance thresholds, similarity measures, algorithm settings) remains insufficient. These choices should be justified, including their potential effect on robustness. | We thank the reviewer for the methodological suggestions.  Regarding parametrization of the algorithms and libraries used in the manuscript, we added in the paper the following part:  “…*The analysis was performed using the Python Scikit-learn library, specifically the Spectracl Bi-clustering using the default settings, with the “scale” method for row/column normalization. This setting applies two different normalizations one over rows and another over columns, a choice justified by assuming that the variability internal to the indicators could be different of the variability along the regions. Finally, the K-Means methods have the only parameter n_clusters = 3. Before applying any clustering or bi-clustering algorithm data of each indicator have been transformed with z-score to have comparable scale for each variable. This to avoid the pre-dominance of some indicator given its unit of measure*….”  (See section **2. Materials and Methods – 2.1 Biclustering technique**) |
|  | The most critical weakness is that cluster differences are presented only descriptively. To strengthen the validity of the results, they should be supported with **statistical tests**. For example:   - Use **ANOVA** or **t-tests** to demonstrate whether clusters differ significantly on SDG3 indicators. - Apply **post-hoc tests** (e.g., Tukey’s HSD) to identify which clusters differ. - Report effect sizes and p-values to establish the meaningfulness of the clusters.   Without such validation, it is unclear whether the clusters are statistically distinct or artifacts of the algorithm. | We approved this comment and integrated the paper as follows: “….*We used the ANOVA f-test to assess for significant differences between cluster means. The results of these tests, detailed in the Supporting Information (Table S2, S3), provide a clear comparison:* *- K-Means divides regions based on all health indicators, creating groups with similar means and standard deviations. In fact, the algorithm works by minimizing the within-cluster variance (inertia) by finding the centroids of data points using Euclidean distances [59].* *- Bi-clustering, instead, by allowing for partial expression of variables, is able to separate not only Italian regions (rows), but also SDG3 health indicators (columns) making it feasible to create more independent clusters where independence can be seen (at least partially) with ANOVA tests and the Tukey-HSD group test.* *In summary, statistical tests show that Bi-clustering builds better separation, and (partial) cluster independence (see for more details Table S4- S7 in the Supporting Information, where cluster 0 is different from cluster 1 and 2, but cluster 1 is not independent from cluster 2, with ANOVA and Tukey-HSD).  Conversely, K-Means tends to rearrange members in order to have a smaller within - cluster variance (inertia) and a general smaller in-cluster variance renouncing to have independent clusters.* ….. (See section **2.** **Materials and Methods - 2.1 Bi-clustering technique**). Morevover, we performed the post-hoc Tukey HSD test (S6-S7) following the ANOVA test (S4-S5) for independent samples to each bi-cluster and to each cluster obtained with kMeans. We used these tests to study the independence of the clusters in both cases. The Null hypothesis for Anova test being that the means of all the 3 groups are equal (i.e., there's no difference between the groups). While for Tukey HSD the null hypothesis being that the means of the two groups being compared are equal i.e. Tukey works by comparing pairs and not the full groups. While the full statistics are shown in the tables S4 to S7 (see Supporting Information), for the convenience of the reader, the summary of the p-values of the Tukey HSD, for bi-clustering and for kMeans is shown in the Table below (see table S6-S7 in the Supporting Information for the full version): **Clusters** \| **BiClustering** \| **Kmeans**; **(0, 1)** \| 0.024** \| 0.929; **(0, 2)** \| 0.001*** \| 0.999; **(1, 2)** \| 0.222 \| 0.975 Each pair of clusters 0,1 or 0,2 has the corresponding p-value for the test statistics in the two cases BiClustering and kMeans. When the p-value is lower than 0.05 we can conclude that the two clusters have a significant difference in the individual means. In synthesis for the bi-clustering we obtain significant differences in the combinations 0,1, 0,2, 1,0, 2,0 but not in the case 1,2 and 2,1 clusters. Both tests (ANOVA and Tukey-HSD) confirm the results with p-values that are lower than 0.05 except for the 1,2 pair of clusters. Instead, in the case of K-Means for both tests (ANOVA and Tukey-HSD) there is no independent set of clusters as the p-value is always (much) larger than 0.05. There is likely much more relevance and information in the bi-clusters rather than in the k-Means clustering, a confirmation that the partial expression is able to recover non obvious patterns in the dataset. We conclude that one of the strengths of the bi-clustering is its ability to get patterns that, being expressed partially, could be less noisy and more clear to reveal. (See **Supporting Information)** |
| **Motivation** | The rationale for using bi-clustering remains weak. Please clarify why this method was chosen over conventional techniques (k-means, hierarchical clustering, PCA) and what unique insights it offers in the SDG3 context. | Thanks for the suggestion. We clarify this point, integrating the paper as follows:  “…..*bi-clustering is not just a novel application; it is a promising methodological tool that generates more robust and statistically reliable findings because it overcomes limitations inherent in both traditional econometric methods (like panel analysis) and standard unsupervised clustering (like k-Means), making it a promising tool for regional-level research. Specifically, compared to standard panel data methods, bi-clustering offers major robustness by avoiding restrictive assumptions relative to:*   - *Assumption-Free Analysis: Bi-clustering requires no assumptions regarding linearity, independence, or inner correlation of the data. This eliminates the necessity for complex diagnostic testing (such as those for collinearity or endogeneity) that often complicates and biases estimations in econometric panel models.* - *Time-Insensitivity: It is highly effective in contexts where the time dimension is insufficient for reliable panel analysis, or where the research focus is primarily on spatial heterogeneity rather than temporal dynamics.*   *Moreover, bi-clustering significantly enhances pattern discovery by leveraging the concept of “partial expression,” which is crucial when variables are numerous, and observations are relatively scarce (both spatially and temporally) as follows:*   - *Bidimensionality for Less Noise: Traditional methods (like k-Means) create unidimensional clusters (grouping regions based on all indicators), forcing relationships to hold across the full dataset. In contrast, Spectral Bi-clustering [55] simultaneously clusters rows (Italian regions) and columns (SDG3 indicators). This bi-dimensionality ensures that bi-clusters only include SDG3 indicators that are more similar and constant (low variance) within that specific subset of Italian regions.* *This focused approach systematically reduces noise and maximizes pattern coherence.* - *Capturing Localized Phenomena: This ability to discover “partial expressions” is key for regional research. A variable (e.g., an SDG3 health indicator) may be highly relevant in a subset of Italian regions, but not across the entire Italy. Bi-clustering inherently captures these localized relationships (e.g., specific regional dependencies, like border regions benefiting from foreign health facilities) that would otherwise be masked by national averages or require the manual addition of complex dummy variables in traditional models…..”*   (See section **2.** **Materials and Methods - 2.1 Bi-clustering technique**). |
| **Literature review** | The literature review has been improved but still does not clearly define the research gap. Please explicitly state what prior studies have done, where they fall short, and how your study differs. | According to this comment, the paper was integrated as follows:  “….*Evidence on SDG implementation points to difficulties in coordination and integration and highlights the absence of operational guidance for translating global goals into subnational action plans: goals are often viewed as too broad, focusing on national and global averages without accounting for significant within-country distributional inequalities [14,15,16].*….  *….* *much of the empirical assessment of SDG 3 progress has concentrated on national scores or cross-country comparisons which—though informative—mask within-country heterogeneity crucial for designing appropriate policies [11,12,17]. Current SDG 3 monitoring efforts largely aggregate indicators to country means or produce dashboards that do not systematically identify “where” and “which” targets are underperforming within countries [4,10,17].*  *In practical terms, policymakers need evidence and methods to (i) identify groups of territories that share similar challenges in achieving SDG 3 targets, and (ii) isolate the indicators most responsible for underperformance within each group. Prior EU-wide and national case studies either (a) remain at the national scale, or (b) describe subnational disparities without pinpointing the subset of SDG 3 indicators that most strongly differentiate such groups [17,19]. This leaves a clear research gap: subnational, indicator-sensitive analyses are needed to go beyond averages and to reveal “which indicators matter, where” in a way that is directly actionable for regional planning. This need is particularly acute in countries with decentralized health systems and marked regional differences [22,23,24,25,26,27,28].* ….  …. *it is essential that research examine differences among regional health systems to understand how global objectives such as SDG 3 can be effectively translated into meaningful social change. By monitoring health outcomes at the regional level, policymakers can identify obstacles to SDG implementation and promote effective, equity-oriented policies. In this sense, there is a gap in the literature that this study seeks to fill by offering a subnational perspective that responds to calls to move beyond averages and to identify context-specific barriers and enablers for the implementation of SDG 3 in Italy.*”  **(See section 1: Introduction)** |
|  | Include more recent SSCI/SCI-indexed works and international reports, especially those discussing design–outcome relationships, financing barriers, and local variation. | Based on this feedback, we made the following updates:  “….*Recent Italian and WHO reports—documenting the persistence of territorial gaps in health outcomes—confirm the relevance of regional-scale analyses to support policy development and decision-making appropriate to diverse territorial contexts [31,46]. The recent ISTAT report likewise underscores the centrality of a subnational reading of the SDGs in Italy, particularly Goal 3 [47]. In line with the “no one left behind” principle, it reinforces the focus on territorial inequalities. Regional analysis shows a heterogeneous picture, with generally better performance in the North-East and delays in the South and Islands; despite signs of recent improvement in some southern regions, progress still does not bridge the historical gaps with the North. These findings strengthen the case, in the Italian context, for regional analyses and targeted policies to fully translate SDG 3 into equitable and context-sensitive health outcomes [47].*  *In addition, recent Italian evidence has reinforced the link between the “design” of health services and health outcomes, while highlighting pronounced local variation [48,49]. Studies also point to a non-negligible organizational–geographic component, suggesting that differences in the design and management of regional services (e.g., supply, waiting times) translate into disparities in utilization and, ultimately, outcomes. Finally, evidence on fiscal and organizational decentralization suggests a risk of amplifying pre-existing inequalities in access to, availability and use of services, and in health outcomes, when resources are devolved without a full understanding of regional characteristics, needs, and gaps [50].*  *This recent evidence—centered on design–outcome relationships, financial barriers, and local variation—justifies our subnational approach*……”  **(See section 1: Introduction)**  These are the integrated references:  - Sarti, F. M. (2023). Challenges in assessment of health systems decentralization: the role of path dependence and choice of indicators. *International Journal of Health Policy and Management*, *12*, 01-10. [citation 50]  - Naghavi, M., Zamagni, G., Abbafati, C., Armocida, B., Agodi, A., Alicandro, G., … & Monasta, L. (2025). State of health and inequalities among Italian regions from 2000 to 2021: a systematic analysis based on the Global Burden of Disease Study 2021. *The Lancet Public Health*, *10*(4), e309-e320. [**citation 49**]  - Matranga, D., & Maniscalco, L. (2022). Inequality in healthcare utilization in Italy: how important are barriers to access? *International Journal of Environmental Research and Public Health*, *19*(3), 1697. [**citation 48**]  - World Health Organization. (2024). *Aligning for country impact: 2024 progress report on the Global Action Plan for Healthy Lives and Well-being for All*. World Health Organization. [**citation 46**]  - ISTAT REPORT 2024, available at <https://www.istat.it/wp-content/uploads/2024/12/2024-SDGs-Report-Ebook.pdf> [**citation 47**]  To the best of our knowledge, there are no recent studies sufficiently aligned with the objectives and conceptual framing of our research to warrant integration into the manuscript. |
| **Research question** | The research question is implied but not explicitly stated. Please formulate it clearly (e.g., *“How can bi-clustering of SDG3 indicators reveal distinct groups of countries, and what policy implications follow?”*). | In response to this comment, the manuscript has been revised as follows:  *“….Specifically, we apply an innovative data-mining technique—bi-clustering [51]—to ISTAT’s regional SDG 3 indicators (2013–2019) in order to: (1) identify groups of Italian regions with similar SDG 3 profiles; (2) determine, for each group, which indicators are most salient (i.e., most strongly expressed) and how they deviate from national averages; and (3) translate these findings into cluster-tailored implications for health policy. These aims are articulated in two research questions (RQs):*   1. ***How can bi-clustering of SDG 3 indicators reveal distinct groups of Italian regions, and what insights do these clusters provide about regional health-related trends?*** *The innovation of the bi-clustering algorithm lies in its ability to investigate the nuanced interaction between regions and SDG3 targets. Unlike traditional clustering, which groups regions based on all variables, bi-clustering allows us to examine the “partial expression” of a variable within a specific cluster. This means we can explore the relevance of a particular SDG3 target across different groups of regions, even when that variable does not represent a strong or weak signal in the overall dataset. This capability is crucial for policymakers because it helps pinpoint region-specific factors shaping health outcomes that might otherwise be obscured by national or broad regional averages.* 2. ***Which SDG 3 variables within each cluster deviate most from the national average, and what policy implications follow from these disparities?*** *Addressing this second question enables us to draw direct conclusions about potential barriers to achieving SDG3 within each group of regions (clusters). This is fundamental to the SDG agenda, which emphasizes that no one should be left behind. By identifying these critical deviations, our research provides the evidentiary basis needed to design health policies or strategies that account for the specific constraints of each territory. This enables policymakers to craft genuinely “tailored” interventions aimed at achieving SDG3 targets, moving beyond a standardized approach and promoting genuine health equity.*   **(See section 1: Introduction)** |
| **Abstract** | The abstract is overly long, repetitive, and contains too many technical details. Please rewrite it more concisely, covering aim, method, key findings, and implications. | We revised the abstract as follows:  “*Health equity is a key policy priority in public health and a central component of Sustainable Development Goal 3 (SDG 3), which focuses on “Good Health and Well-Being”. While SDG 3 sets global benchmarks, its local implementation—particularly in countries with decentralized healthcare systems and significant regional disparities—poses a considerable challenge.*  *This study addresses the need for subnational analyses by moving beyond national averages and identifying region-specific barriers and enablers to achieving SDG 3 targets, using Italy as a case study.*  *To this end, we apply spectral bi-clustering, an innovative data-mining technique, to regional SDG 3 indicators compiled by ISTAT for the years 2013–2019. The analysis pursues two objectives: (1) to identify clusters of Italian regions with similar SDG 3 profiles; and (2) to determine which indicators are most salient within each cluster and how they diverge from national benchmarks, deriving policy implications tailored to each group of regions.*  *Our findings reveal three distinct regional clusters: the analysis demonstrates that certain health indicators are more relevant within specific regional contexts, pointing to structural and systemic variations in healthcare provision and outcomes. These results underscore the inadequacy of uniform policy approaches and highlight the need for regionally differentiated strategies.*  *This study provides one of the first applications of spectral bi-clustering to health equity analysis at the subnational level, offering actionable insights for policymakers seeking to localize SDG 3 implementation and bridge health gaps across regions*.”  **(See the Abstract)** |
| **Discussion and Policy Implications** | The discussion includes very general recommendations (e.g., “countries should cooperate”). Recommendations should be **concrete and measurable**, such as:   - Indicating which low-performing countries should prioritize specific SDG3 indicators. | In response to this comment, the manuscript has been revised as follows:  “…*In this sense, concrete and measurable recommendations for regions in this low-performing cluster include: prioritizing (i) reduction of SDG 3.4 risks by lowering standardized diabetes rates from 1.26× to ≤ 1.00× the national average and excess body weight from 1.12× to ≤ 1.00× within 5 years through funded primary care and lifestyle programs, (ii) strengthening the SDG 3.c workforce by increasing the number of nurses/midwives from 0.85× to ≥ 1.00× the national average ( +15%) within 3 years, (iii) restoring ordinary inpatient bed capacity from 0.89× to ≥ 1.00× per 100,000 population within 5–7 years, and (iv) achieving SDG 3.6 by reducing road-traffic fatalities from 1.35× to ≤ 1.00× within 3 years through law enforcement, emergency response networks, and remediation of crash hotspots.*  *For regions in this cluster, progress should be verified annually using the ratio_bc_country metric reported in Table 1*….”  (See **section 4 – Discussion**) |
|  | - Suggesting mechanisms for knowledge transfer from high-performing clusters. | Following this suggestion, we updated the paper as follows:  “…*At the national level, to promote convergence across all regions and reduce divergences among the three clusters, we recommend developing structured knowledge-transfer mechanisms from higher-performing clusters to lagging regions. To this end, it is essential to: (a) design action plans targeted to each region’s priority SDG 3 gaps, (b) implement short, focused placements for clinical, managerial, and public-health staff to transfer routines and governance practices from better-performing regions to those lagging on one or more SDG 3 targets, (c) develop concise, adaptable protocols (prevention, workforce, and service delivery) aligned with local capacities, and (d) conduct recurring reviews of regional cases and learning sessions centered on the indicators showing the largest gaps.*  *Together, these mechanisms convert observed strengths into reproducible routines while maintaining general, transferable, and measurable recommendations against SDG 3 progress*….”  (see **section 4 – Discussion**) |
|  | - Explaining how international organizations might use clusters for resource allocation. | As requested, we integrated the following changes:  “…*In light of this evidence, international organizations can also use clustering to ensure that scientific evidence informs appropriate resource allocation. For example, countries could be grouped by co-occurring needs and delivery constraints, and financial resources could then be distributed so that clusters furthest from target outcomes receive higher per-capita allocations. Finally, the composition of clusters should be monitored annually, with entities reclassified as systems improve*…..”  (See **section 4 – Discussion**) |
| **Diversity of Recommendations** | Current recommendations are one-dimensional. Please tailor them to different stakeholders:   - **Policymakers:** concrete policy actions for low- and middle-income countries. | Accordingly, we have implemented the following integrations:  “…*Our study allows us to offer recommendations for different stakeholders.*  *First, in light of our results, national policy makers should channel greater investments toward initiatives that advance SDG 3, especially in underserved and economically disadvantaged regions, optimizing resource allocation to improve health-service management where needs are most urgent. We also underscore the adverse effects of the RRPs, which, while reducing regional debt, have inadvertently worsened health indicators.*  *The methodology we applied in Italy can serve as a valuable model for monitoring SDGs in other national settings, particularly those with significant internal disparities. For example, countries with decentralized or devolved governance structures—such as India, Brazil, or the United States—could use this method to identify which SDG-specific variables are most critical for their subnational regions. In doing so, policy makers could move beyond national averages and design interventions tailored to local needs*…”  (See **section 5 – Conclusion**) |
|  | - **Researchers:** directions for extending bi-clustering to other SDGs. | Based on this feedback, we made the following updates:  “..*For researchers, the bi-clustering approach is not limited to SDG 3; it can be extended to other indicators to reveal important regional differences in areas such as poverty (SDG 1), education (SDG 4), or access to safe drinking water (SDG 6), thereby supporting appropriate resource allocation and the development of more effective, targeted policies. In practical terms, it will be necessary to: (a) identify the clusters, (b) determine which diverge most from SDG targets and therefore require targeted action, and (c) repeat the analysis over time to observe how clusters change before and after reforms, and whether gaps narrow or widen*..”  (See **section 5 – Conclusions**) |
|  | - **International agencies:** implications for funding and monitoring. | To address this point, we integrated the manuscript as follows:  “…*Finally, we conclude with the following recommendations for international agencies: (a) link funding to subnational clusters rather than national averages, so that resources follow the largest gaps, (b) ask countries to publish simple quarterly dashboards that show each indicator relative to the national average, making progress visible and comparable, and (c) create a learning loop by sharing models, code, and examples, enabling countries to replicate what works and correct what does not at the cluster level*..”  (See **section 5 – Conclusion**) |
| **Originality** | At present, the claim of originality rests mainly on the novelty of applying bi-clustering in this context. This needs to be strengthened by comparing your results more explicitly with prior clustering studies and by showing how your approach contributes **methodologically or theoretically**, not only through application. | We thank the reviewer for this constructive feedback. We integrated the manuscript as follows:  “….*Bi-clustering is not just a novel application; it is a promising methodological tool that generates more robust and statistically reliable findings because it overcomes limitations inherent in both traditional econometric methods (like panel analysis) and standard unsupervised clustering (like k-Means), making it a promising tool for regional-level research.*  *Specifically, compared to standard panel data methods, bi-clustering offers major robustness by avoiding restrictive assumptions relative to:*   - *Assumption-Free Analysis: Bi-clustering requires no assumptions regarding linearity, independence, or inner correlation of the data. This eliminates the necessity for complex diagnostic testing (such as those for collinearity or endogeneity) that often complicates and biases estimations in econometric panel models.* - *Time-Insensitivity: It is highly effective in contexts where the time dimension is insufficient for reliable panel analysis, or where* *the research focus is primarily on spatial heterogeneity rather than temporal dynamics.*   *Moreover, Bi-clustering significantly enhances pattern discovery by leveraging the concept of “partial expression,” which is crucial when variables are numerous, and observations are relatively scarce (both spatially and temporally) as follows:*   - *Bidimensionality for Less Noise: Traditional methods (like k-Means) create unidimensional clusters (grouping regions based on all indicators), forcing relationships to hold across the full dataset. In contrast, Spectral Bi-clustering simultaneously clusters rows (Italian regions) and columns (SDG3indicators). This bi-dimensionality ensures that bi-clusters only include SDG3 indicators that are more similar and constant (low variance) within that specific subset of Italian regions. This focused approach systematically reduces noise and maximizes pattern coherence.* - *Capturing Localized Phenomena: This ability to discover “partial expressions” is key for regional research. A variable (e.g., an SDG3 health indicator) may be highly relevant in a subset of Italian regions, but not across the entire Italy. Bi-clustering inherently captures these localized relationships (e.g., specific regional dependencies, like border regions benefiting from foreign health* *facilities) that would otherwise be masked by national averages or require the manual addition of complex dummy variables in traditional models*….”   (See section **2. Materials and Methods – 2.1 Biclustering technique**) |
