# Supplementary material for: Unveiling and understanding health inequalities: A bi-clustering study on SDG3 implementation in the Italian regions
Source: PLoS One. 2026 Mar 26;21(3):e0340438. doi: 10.1371/journal.pone.0340438 (PMC13020981; doi:10.1371/journal.pone.0340438)
Supplement: Attachment — Submitted filename: Response_to_Reviewers_auresp_3.docx [file pone.0340438.s016.docx]

**Authors’ response to Reviewers’ comments**

**MANUSCRIPT NUMBER: PONE-D-25-29410R2**

**Unveiling and Understanding Health Inequalities: A Bi-Clustering Study on SDG3 Implementation in the Italian regions**

The authors of this paper would like to thank the reviewers of PLOS ONE for their insightful comments and constructive suggestions, which have helped us significantly improve the quality and clarity of our manuscript. Please note that the revised manuscript has been submitted in Track Changes mode (Revised Manuscript with Track Changes), allowing you to easily identify all modifications made in response to the reviewers’ feedback. Additionally, a clean version of the manuscript has been submitted under the filename “Manuscript”.

|  | **Reviewer #2’s comment** | **Authors’ responses** |
| --- | --- | --- |
| **Methodology -** Reporting of ANOVA results | The methodology section states that “post-hoc tests were performed (one-way ANOVA, p < 0.05)”; however, the results are not shown in the tables. It is recommended that the authors add basic statistics such as the F-value, p-value, and effect size (η²) for each cluster to the table or present them as supplementary material. This would allow readers to verify the statistical validity of the cluster differences. | We thank the reviewer for the methodological suggestions.  Basic statistical values are detailed in the Supporting Information: the **F-value** and **p-value** are presented in the Table S4 and S5, while the **effect size** is reported in the Table S8.  The text was integrated as follows:  “…*We used the ANOVA f-test to assess for significant differences between cluster means (Table S4, S5 in the Supporting Information show F-value and p-value). In addition, we performed, for the same goal, the Kruskall-Wallis test to provide an alternative comparison (Table S2, S3 in the Supporting Materials)*…  …. *Moreover, the K-Means methods have the only parameter n_clusters = 3. We remember that before applying any clustering or bi-clustering algorithm data of each indicator have been transformed with z-score to have comparable scale for each variable. This to avoid the pre-dominance of some indicator given its unit of measure. Finally, we investigated the “size effect” of each cluster by computing the Cohen’s d effect size measure for bi-clustering and k-means. Based on this metric, the bi-clustering results yield stronger distinctions: all bi-clusters demonstrated at least a medium effect size, with clusters 0 and 2 achieving a larger effect size. Conversely, all k-Means clusters show a small effect size, indicating that their group averages are too similar to indicate a meaningful difference (Table S8 in the Supporting Materials)*..”  (See section 2.1 Bi-clustering technique) |
