# Supplementary material for: Unveiling and understanding health inequalities: A bi-clustering study on SDG3 implementation in the Italian regions
Source: PLoS One. 2026 Mar 26;21(3):e0340438. doi: 10.1371/journal.pone.0340438 (PMC13020981; doi:10.1371/journal.pone.0340438)
Supplement: Attachment — Submitted filename: Reviewer Comment.docx [file pone.0340438.s011.docx]

**Unveiling and understanding health inequalities: a Bi-Clustering study on SDG3 implementation in the Italian region**

**Originality**

The manuscript provides a notable degree of originality by applying the **Spectral Bi-Clustering** method to analyze subnational health inequalities in the context of **SDG3 (Good Health and Well-Being)**. This technique is rarely used in health systems research and introduces the concept of **“partial expression”** to explore the interaction between regions and health indicators—adding substantial methodological value.

**However:** To further emphasize its originality, the manuscript should include a clearer discussion of prior studies (if any) that have used similar methods in public health, highlighting how this study offers a novel application.

**Methodology**

The use of **Spectral Bi-Clustering** enables multidimensional analysis and allows for simultaneous clustering of both regions and indicators—an advantage over traditional one-dimensional clustering.

**Recommendations for improvement:**

- The methodological section would benefit from more technical detail, such as how normalization, variance filtering, or missing data handling were conducted.
- The advantages of bi-clustering over other methods (e.g., k-means, PCA) should be demonstrated with a concrete example.
- Details on software or libraries used (e.g., Python, R, Scikit-learn) should be specified.

**Alignment with the Topic**

The manuscript’s title aligns well with its content. The focus on SDG3 and health inequalities in the Italian context is relevant, timely, and fits within the scope of international health systems and policy journals.

**1. Abstract**

**Strengths:**

- The abstract clearly defines the topic, method, and main findings.

**Suggestions:**

- The final sentences should explicitly state the **study’s contribution to the field**, such as: “This study is among the first to apply spectral bi-clustering in regional health equity analysis.”
- A short sentence on the **policy implications** would strengthen its relevance and appeal.

**2. Introduction**

**Strengths:**

- Provides a solid rationale for the study.
- Research questions are clearly articulated.

**Suggestions:**

- The **research gap** should be emphasized more explicitly.
- The **significance of the research questions** could be elaborated—why are they important for policymakers or the SDG agenda?

**3. Literature Review / Background**

**Strengths:**

- Comprehensive overview of health equity, SDG3, and Italian regional disparities.
- Solid contextual foundation.

**Suggestions:**

- A clearer comparison with **existing bi-clustering applications** in public health is needed to justify the choice of method.
- A **brief mention of how SDG3 has been addressed in other countries** could situate Italy’s case within a broader global context.
- The **literature gap** should be explicitly stated and linked to the research questions.

**4. Methods**

**Strengths:**

- Clustering process and rationale for three-region categorization are clearly presented.

**Suggestions:**

- Details regarding **data pre-processing** (e.g., normalization, missing data, outliers) should be added.
- The **statistical significance of cluster differences** (e.g., ANOVA, t-tests) is not discussed—this would enhance methodological rigor.
- Software used and parameter settings should be described.
- A clearer justification for the selection and inclusion/exclusion of SDG3 indicators would be helpful.

**5. Discussion**

**Strengths:**

- The discussion links findings to regional policy and institutional structures.
- Highlights how decentralization and austerity policies have shaped health disparities.

**Suggestions:**

- **Comparison with previous studies** is limited. The discussion would be stronger if the findings were contrasted with existing literature.
- The **limitations of bi-clustering** (e.g., sensitivity to initial conditions, lack of time series integration) should be discussed explicitly.
- The exclusion of 2020–2021 data due to COVID-19 is justified, but its implications on generalizability should be acknowledged.

**6. Conclusion**

**Strengths:**

- Summarizes key findings and policy recommendations effectively.

**Suggestions:**

- The **theoretical and methodological contributions** of the study should be clearly stated.
- A short reflection on the **utility of bi-clustering for SDG monitoring** in other national contexts would add depth.
- The **future research directions** section could be expanded—for example, exploring SDG indicators beyond SDG3, or applying this approach in other decentralized health systems.

**Final Remarks and Recommendation**

This manuscript makes a valuable and original contribution to the literature on regional health inequalities and SDG implementation. However, addressing methodological clarity, enhancing the literature discussion, and articulating the study’s limitations will significantly improve the paper’s scientific robustness and publication potential.
